# Headway made towards biosignatures for incipient tuberculosis

**DOI:** 10.1016/S2213-2600(19)30355-8

**Published:** 2020-01-17

**Authors:** Thomas J Scriba, Simon C Mendelsohn

**Affiliations:** South African Tuberculosis Vaccine Initiative, Institute of Infectious Disease and Molecular Medicine and Division of Immunology, Department of Pathology, University of Cape Town, Cape Town 7925, South Africa

Identification of individuals with tuberculosis for antibiotic treatment is a major component of the of WHO’s End TB Strategy.^1^ Diagnostic tests for tuberculosis rely on detection of *Mycobacterium tuberculosis* in sputum expectorated by a person with productive cough. The search for new sputum-independent diagnostic tests is gaining momentum, as exemplified by advances in urine-based point-of-care lipoarabinomannan lateral flow assays for use in hospitalised patients with HIV.^2^

Active case-finding strategies are crucial for reducing transmission and achieving population-level tuberculosis control.^3^ A non-sputum-based triage test that can identify individuals with undiagnosed disease for further clinical investigation could shorten the time to diagnosis and treatment. The response to this need has included extensive development of blood transcriptomic signatures as diagnostic or screening tests for tuberculosis.^4^

Identification of asymptomatic individuals with subclinical and so-called incipient tuberculosis, who are at high risk of progression to active disease, has also gained momentum. However, available tests for *M tuberculosis* infection, the tuberculin skin test and interferon-γ release assay (IGRA), perform poorly as predictors of disease progression, with pooled positive predictive values of less than 3%.^5^ Blood transcriptomic incipient tuberculosis signatures could have better accuracy^6^ and might allow targeted therapy to prevent disease before symptoms emerge and potentially stop transmission of *M tuberculosis*. Mathematical modelling suggests that a targeted preventive therapy strategy using IGRA at 30% screening coverage in South African adults without HIV infection could reduce tuberculosis incidence in 2035 by 39% (95% CI 31–48).^7^ By comparison, a transcriptomic biomarker for incipient tuberculosis would reduce incidence by 20% (15–27).^7^ However, more individuals would have to be treated per tuberculosis case averted with the IGRA (84 [59–123]) than with an incipient tuberculosis biomarker (49 [29–77]).^7^ To maximise their effect, such strategies could be employed for mass screening in tuberculosis-endemic communities and targeted screening in high-risk groups, such as contacts of tuberculosis patients, previous tuberculosis cases, migrants, the homeless, HIV-infected, diabetic, and immunosuppressed individuals, prison inmates, and individuals residing in high density peri-urban slums.

In *The Lancet Respiratory Medicine*, Rishi Gupta and colleagues^8^ applied 17 published tuberculosis diagnostic and predictive mRNA signatures to pooled transcriptomic datasets from four published, longitudinal studies. Eight concise signatures differentiated samples with incipient tuberculosis from controls with very similar accuracy over a 2-year period. None of the signatures met the minimum performance criteria for an incipient tuberculosis test defined in a WHO target product profile,^9^ except when measured within 3 months of diagnosis. Waning transcriptomic signature accuracy when tested more than 3 months before diagnosis, but detection of signature positivity in some individuals up to 24 months before disease diagnosis, is consistent with the heterogenous duration of disease progression and the dynamic course of incipient and subclinical tuberculosis disease states between individuals. Disease progression and regression are also likely to occur in infected individuals,^10^ reducing the sensitivity and specificity of any test for disease progression. Given the spectrum of incipient and subclinical tuberculosis disease suggested by these scenarios, we question the achievability of a predictive test with sensitivity and specificity of more than 90% over a 24-month time horizon that are outlined as the optimal criteria in the target product profile.

The study by Gupta and colleagues^8^ informs rational selection of transcriptomic signatures for further development towards a point-of-care test. It also highlights the paucity of longitudinal cohort studies with incident tuberculosis cases on which analyses of incipient and subclinical tuberculosis biomarkers depend. The next step is assessment of the best performing biomarkers in prospective field studies that enrol participants without the stringent inclusion and exclusion criteria typically used in case-control studies, which might lead to selection biases and exaggerated test performance. Transcriptomic signature evaluation should also include individuals with previous tuberculosis disease, HIV infection, diabetes mellitus, and malnutrition and children and individuals from wider geographical distributions, to assess how these variables affect biomarker performance. Although RNA sequencing and microarrays are necessary for discovery, application of these signatures at the point-of-care will require cheaper and more tractable technologies. The Correlate of Risk Targeted Intervention Study (NCT02735590) addresses these points. Results from this randomised controlled trial of isoniazid and rifapentine therapy to prevent pulmonary tuberculosis in high-risk individuals identified by a PCR-based, transcriptomic signature are anticipated in early 2020.

Feasibility and cost are major implementation hurdles for any biomarker and realisation of a mass screen-and-treat preventive strategy for tuberculosis will require new technologies that are able to process samples rapidly and at high throughput at the point of care. Targeted preventive therapy could have a substantial impact on tuberculosis incidence. Time will tell if host-response biomarkers of incipient tuberculosis could be incorporated into the WHO End TB Strategy.
